# Helmet Phthalocyaninato Iron Complex as a Primary Drier for Alkyd Paints

**DOI:** 10.3390/ma14051220

**Published:** 2021-03-05

**Authors:** Jan Honzíček, Eliška Matušková, Štěpán Voneš, Jaromír Vinklárek

**Affiliations:** 1 Institute of Chemistry and Technology of Macromolecular Materials, Faculty of Chemical Technology, University of Pardubice, Studentská 573, 532 10 Pardubice, Czech Republic; eliska.matuskova@student.upce.cz (E.M.); stepan.vones@student.upce.cz (Š.V.); 2 Department of General and Inorganic Chemistry, Faculty of Chemical Technology, University of Pardubice, Studentská 573, 532 10 Pardubice, Czech Republic; jaromir.vinklarek@upce.cz

**Keywords:** alkyd paints, iron, phthalocyanine, primary drier, infrared spectroscopy

## Abstract

This study describes the catalytic performance of an iron(III) complex bearing a phthalocyaninato-like ligand in two solvent-borne and two high-solid alkyd binders. Standardized mechanical tests revealed strong activity, which appeared in particular cases at concentrations about one order of magnitude lower than in the case of cobalt(II) 2-ethylhexanoate, widespread used in paint-producing industry. The effect of the iron(III) compound on autoxidation process, responsible for alkyd curing, was quantified by kinetic measurements by time-resolved infrared spectroscopy and compared with several primary driers. Effect of the drier concentration on coloration of transparent coatings was determined by UV–Vis spectroscopy.

## 1. Introduction

Coordination compounds bearing phthalocyanine and phthalocyanine-like ligands represent an important class of transition metal complexes because they are structurally related with naturally occurring porphyrin species. Due to thermal stability, large-scale availability, and lower cost, they have found several applications in the fields of material science [1] and catalysis [2].

**Figure 1:**
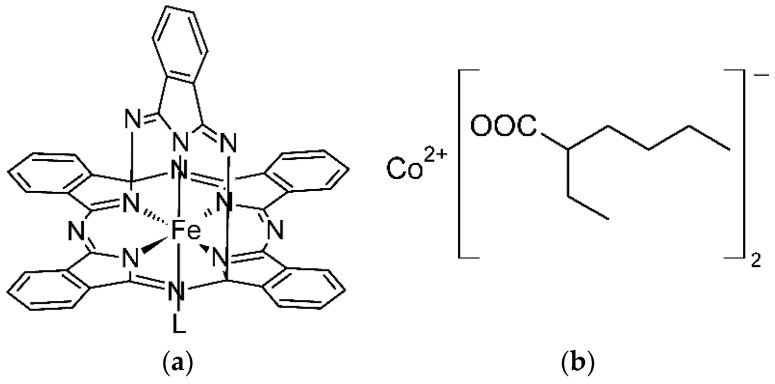
Chemical formulas of: (**a**) phthalocyaninato iron(III) complex (Fe-diiPc); (**b**) cobalt(II) 2-ethylhexanoate (Co-2EH).

In 2006, McGaff et al. described an iron(III) complex bearing bicyclic ligand derived from phthalocyanine with a helmet-like structure ([Fe(diiPc)L]; Fe-diiPc, Figure 1a), where diiPc is the formally trianionic 14,28-(1,3-diiminoisoindolinato)phthalocyaninato ligand and L represents the labile axial ligand (e.g., MeOH, H_2_O) that completes the coordination sphere of iron. This compound has a unique molecular structure with a non-planar phthalocyanine core, as documented by X-ray diffraction analysis [3]. Five nitrogen donor atoms of the diiPc ligand enforce very specific geometric constrains in the coordination sphere of iron, mimicking the active site of lipoxygenases, non-heme iron-based enzymes, responsible for the peroxidation of linoleate, linolenate and arachidonate [4]. It is noteworthy that only few related phthalocyanine complexes with helmet-like structure have been reported. They include isostructural cobalt(III) compound [Co(diiPc)L] [3] and congeners of Fe-diiPc modified in the ligand periphery [5,6].

The parent compound, Fe-diiPc, provides promising catalytic properties, as documented in studies dealing with the epoxidation of olefins [5], oxidation of cycloalkanes [7], and oxidation of primary and secondary alcohols [6,8,9]. Catalytic effect of Fe-diiPc on the oxidative drying of sunflower fatty acid methyl esters have been described [10]. Such systems serve as a simplified model for air-drying binders, commonly used in paint-producing industry due to a low price and a medium to high content of renewable feedstocks [11]. Alkyd resins, used in this study, represent the most widespread class of the air-drying binders; they are polyesters of saturated dicarboxylic acids and polyalcohol modified with fatty acids of semidrying or drying plant oils [12]. Chemical curing of the alkyd resins proceeds in the polyunsaturated fatty acid tails by the action of air-oxygen. This process, known as autoxidation, proceeds at room temperature but very sluggishly when not promoted by additives known as primary driers [11].

Our research of coordination compounds of redox-active transition metals is motivated by the recognized carcinogenic properties of soluble cobalt compounds [13], currently used as the primary driers, and a limited portfolio of cobalt-free alternatives, because none of them fully fits the demands of the paint-producing industry [11]. We note that widespread use of cobalt(II) 2-ethylhexanoate (Co-2EH, Figure 1b) is still acceptable for common paints, but the situation might change rapidly due to the ongoing tightening of legislative restrictions [14]. Commercial cobalt-free systems usually involve manganese(II) carboxylates [15] or their mixtures with *N*-chelators (e.g., bipyridine) [16], but these systems suffer from discoloration and a low activity at adverse climatic conditions [15]. Promising drying characteristics are further reported for manganese(III) acetylacetonate [17], manganese complexes of 1,4,7-trimethyl-1,4,7-triazacyclononane [14], oxidovanadium(IV) carboxylates [18,19], oxidovanadium(IV) acetylacetonates [20,21,22], and oxidovanadium(V) dithiocarbamates [23]. The field of iron-based primary driers is currently focused mainly on commercial iron bispidine complex, known under the trademark Borchi OXY-Coat, which performs at very low concentrations compared to Co-2EH [24,25], and its congeners with increased solubility in organic solvents [26,27]. Considerable attention has further been given to iron complexes with polydentate *N*-chelators [14] and ferrocene derivatives bearing electron-withdrawing substituents [28,29]. The activity of monocyclopentadienyl compounds [(η^5^-C_5_H_5_)Fe(CO)_2_I], [{(η^5^-C_5_H_5_)Fe(CO)}_2_(μ-CO)_2_] and [(η^5^-C_5_H_5_)Fe(η^5^-C_6_H_7_)] has been reported in patent literature [30].

The aim of this study was to investigate the curing of selected solvent-borne and high-solid alkyd resins by the action of Fe-diiPc. Catalytic activity of this species will be determined at room temperature by standardized mechanical tests. Chemical transformations in the alkyd binders will be followed be infrared and Raman spectroscopy. We note that Fe-diiPc was chosen based on promising results of preliminary testing on model systems and one-step synthetic procedures from readily available starting compounds [10], which fulfills our long-term goal to reduce the cost and environmental impact of primary driers for room temperature curable air-drying paints.

## 2. Materials and Methods

The commercial driers cobalt(II) 2-ethylhexanoate (Co-2EH; 65 wt.% in mineral spirits) and Borchi OXY-Coat (0.0846 wt.% of metal) were obtained from Sigma-Aldrich (St. Louis, MO, USA) and OMG Borchers GmbH (Langenfeld, Germany), respectively. The phthalocyaninato iron(III) complex (Fe-diiPc; 3.05 wt.% of metal) was prepared according to the procedure described elsewhere [10]. Alkyd resins CHS-ALKYD S471 X 60 (S471; oil length 47%, dry content 59%), CHS-ALKYD S622 N 60 (S622; oil length 62%, dry content 59.4%) and CHS-ALKYD TI 870 (TI870; oil length 87%, dry content 98.6%) were supplied by Spolchemie (Ústí and Labem, Czech Republic). NEBORES FP 07-90 D (FP07; oil length 68%, dry content 89%) was supplied by Safic-Alcan (Brno, Czech Republic). Dimethyl sulfoxide g.r. (DMSO; C_2_H_6_OS), toluene g.r., used dissolution/dilution of driers, and methylethylketone g.r. (MEK), used for the chemical resistance tests, were supplied by Lach-Ner (Neratovice, Czech Republic). Thinner S 6006 Aroma-free was obtained from Severochema (Liberec, Czech Republic) and used as a thinner for high-solid alkyd resins. Chloroform g.r. (PENTA; Chrudim, Czech Republic) was used as a degreaser for substrates.

### 2.1. Preparation of Test Formulations

Alkyd formulations containing the given driers were prepared in the concentration range 0.1–0.01 wt.% (Co-2EH) or 0.01–0.001 wt.% (Fe-diiPc). Calculated amounts of Co-2EH were treated with 100 µl of toluene for 5 g of alkyd resin. The formulations were stirred vigorously with a spatula to produce a homogenous solution and then degassed in an ultrasonic bath. A similar procedure was used for the preparation of formulations containing Fe-diiPc. In this case, the drier was dissolved in DMSO (100 µL) instead of toluene. Formulations of high solid resin TI870 of high dry matter (98.6%) were diluted by Thinner S 6006 Aroma-free to 90 wt.% of solid content in order to reduce viscosity.

### 2.2. Measurements of Drying Time

Prepared formulations of solvent-borne resins were applied on glass strips (305 mm × 25 mm × 2 mm), cleaned and degreased by chloroform. A frame applicator with a 76 µm gap was used for the spreading the formulations. The drying performance was determined on a B. K. Drying Time Recorder (BYK-Gardner, Geretsried, Germany) according to ASTM D 5895-20 [31] under standard laboratory conditions (temperature 23 °C, relative humidity 50%). Test glass strips were placed into holders of the recorder. The hemispherical-ended needles (D = 1 mm) were placed at the beginning of the film. The experiments were conducted in 24 h mode with a weighted (5 g) needle. The evaluation of set-to-touch time (τ_1_), tack-free time (τ_2_), dry-hard time (τ_3_) and dry-through time (τ_4_) is described in detail in our previous work [17]. Two independent measurements were performed for each formulation; averaged values are reported.

### 2.3. Determination of the Film Hardness

Test coatings applied on glass plates (200 mm × 100 mm × 4 mm) were used for determination of the film hardness. Plates were degreased by chloroform and coated with a frame applicator of a 150 µm gap for solvent-borne and of a 90 µm gap for high-solid alkyd binder. Film hardness development was monitored for 100 days using a Pendulum Hardness Tester (Elcometer, Manchester, UK) with a Persoz type of pendulum according to ISO 1522:2006 [32]. The measurements were performed under standard laboratory conditions (temperature 23 °C, relative humidity 50%). This method is based on registering the number of pendulums swings it takes before the amplitude of the pendulum is damped to a certain extent [32]. The measured values were related to the hardness of a glass standard and expressed as relative hardness. Each sample was measured three times on different positions; averaged values are reported. The error in determination of the hardness was estimated to be ±0.5%.

### 2.4. MEK Resistance and Dry Film Thickness

The chemical resistance and thickness of dry films were measured after 105 days since application on glass plates (200 mm × 100 mm × 4 mm). The test of chemical resistance toward MEK was done by cotton swab, dipped in MEK every 10 s. The swab was rubbed on the surface of the film until the substrate was exposed according to ASTM D 4752 [33]. A Roughness Meter TQC-SP1560 (TQC, Hilden, Germany) was used to measure the dry thickness according to ISO 2808:2007 [34]. Each sample was tested three times on different positions; averaged values are reported. The margin of error for MEK resistance was ±4% and for dry thicknesses ±5%.

### 2.5. Infrared and Raman Spectroscopy

Measurements of vibration spectra were performed on a Nicolet iS50 FTIR spectrometer equipped with Raman module (Waltham, MA, USA). Infrared spectra were measured using a built-in attenuated total reflection (ATR) crystal in region 4000–400 cm^−1^ (64 scans per spectrum, data spacing = 0.5 cm^−1^) under standard laboratory conditions (temperature 23 °C, relative humidity 50%). Raman spectra were measured using Nd:YAG excitation lasers (λ = 1064 nm, power = 0.5 W, 256 scans per spectrum, data spacing = 1 cm^−1^) in the region 3500–200 cm^−1^. The fresh binders were characterized after the evaporation of volatiles, which occurred at room temperature and normal pressure. The binder was applied on the clean glass plates (200 mm × 100 mm × 4 mm) using a frame applicator of 150 µm gap and left for about 30 min. After that, the sample was taken from the glass plates and measured. The samples of cured formulations were taken from the coatings used for the determination of the film hardness as described before. The characterization of the cured samples was performed 105 days after application on glass substrates.

Time-resolved infrared spectra, used for the kinetic studies, were recorded every 5 min for 20 h. The collected spectra were integrated using a fixed two-point baseline in the region 3025–2990 cm^−1^ to estimate intensity of ν_a_(*cis*-C=C–H) band. The rate coefficients (*k_max_*) at the beginning of the autoxidation process were estimated as the steepest slope of the logarithmic plots of the integrated area vs. time. Induction time (*t*_ind_) and autoxidation half-life (*t*_1/2_) values were processed from the original graph in logarithmic and linear scales, respectively.

### 2.6. Measurements of Film Coloration

The UV–Vis spectra of the transparent test coatings were collected on a UV–VIS Maya 2000 Pro spectrometer (Ocean Optics, Dunedin, FL, USA) using halogen and deuterium light sources of a DH-2000-BAL (Ocean Optics). The transmission spectra were processed in OceanView software and expressed in the CIELAB color space with a standard illuminant “D65” and an observer at “2-degrees”. The test formulations of Fe-diiPc/S471 and Co-2EH/S471 were spread over microscopic glass slides (76 mm × 26 mm × 1 mm) using the frame applicators of a 200 µm gap and left for three days under standard laboratory conditions (temperature 23 °C, relative humidity 50%) at a diffuse daylight illumination. All reported data are given relative to the pure microscopic slides. Each sample was measured on six spots; averaged values are reported.

## 3. Results and Discussion

### 3.1. Mechanical Tests on Test Coatings

Determination of the catalytic power of Fe-diiPc was examined in solvent-borne (S471 and S622) and high-solid alkyd binders (FP07 and TI870) using standardized mechanical assays on test coatings applied on glass substrates. They included the determination of drying times by the Beck–Koller method [31], determination of the relative hardness by Persoz pendulum [32], and determination of chemical resistance by the MEK test [33]. The collected experimental data were compared with reference data obtained for coatings treated by the commercial primary drier Co-2EH.

Unusually high catalytic activity of Fe-diiPc was documented on short total dry times (τ_4_), observed for solvent-borne formulations (Table 1). In case of the medium oil-length alkyd resin S471, low values of τ_4_ were obtained at 0.003–0.01 wt.% of metal in dry matter content. In this concentration range, tack-free time (τ_2_) of the formulations Fe-diiPc/S471 decreased with the increasing concentration. Inverse dependence, observed for τ_4_, was due to the formation of a crosslinked polymeric layer on the coating surface at high metal concentration, which is responsible for slower oxygen diffusion and pure through-drying, as previously documented on formulations of Co-2EH [25]. The metal concentration 0.003 wt.% seems to be the optimal dosage for Fe-diiPc in the absence of secondary driers; these can strongly influence the process of through drying. Commercial Co-2EH, used as the reference, shows optimal performance at considerably higher dosage (0.1 wt.%), which is in line with recommendation of suppliers and literature data [17]. At this concentration of Co-2EH, τ_2_ is comparable but τ_4_ is significantly longer than that observed for Fe-diiPc at 0.003 wt.%. Comparison with commercial cobalt-free primary drier Borchi OXY-Coat is given in Table S1 in the Supplementary Materials. Such a compound is highly active at concentrations even lower than Fe-diiPc (0.003–0.0003 wt.%). We note that the inverse dependence of τ_4_ is observed here as well.

Formulation of long-length alkyd resin S622, treated by Fe-diiPc, exhibits rapid drying at a concentration of 0.01 wt.%, as evident from low values of tack-free time (τ_2_ = 0.9 h) and total dry time (τ_4_ = 3.8 h). Lowering of metal concentration leads to the prolongation of drying times. Nevertheless, even at 0.003 wt.%, performance of Fe-diiPc is still acceptable because τ_4_ is shorter than that observed for formulations treated with Co-2EH.

Long-term activity of Fe-diiPc in the test coatings was evaluated by the measurements of their relative hardness. Table 1 summarizes the data obtained for solvent-borne resins S471 and S622. The values obtained for the formulations Fe-diiPc/S471 ten days after application (*H*_rel;10d_) are comparable to Co-2EH/S471. Lower final hardness (*H*_rel;100d_) of the coatings treated by Fe-diiPc, measured one hundred days after application, is attributed to a lower density of crosslinking. Data collected for the binder of longer oil-length (S622) show similar trends. In this case, however, significantly slower hardening of the coatings treated by Fe-diiPc is evidenced already on values of *H*_rel;10d_.

Fe-diiPc exhibited a very good performance in the high-solid binder FP07 at metal concentration 0.01 wt.%, as evident from the short tack-free time (τ_2_ = 3.9 h) and dry-hard time (τ_3_ = 8.3 h); see Table 2. Lowering of the concentration led to prolongation of the drying times, but they were still acceptable at 0.006 wt.%. It should be noted that total-dry time is less important in the case of high-solid binders, owing to their generally purer through-drying and lower relative hardness, which often leads to values higher than 24 h. The optimal dosage of Co-2EH is 0.06 wt.% due to its acceptable dry-hard time (τ_3_) and very low values of τ_1_ and τ_2_. Inverse dependence of τ_3_ on concentration, observed for Co-2EH, suggests considerably worse through-drying at high metal concentration than in the case of Fe-diiPc.

Formulations Fe-diiPc/TI870 were cured slower than the aforementioned Fe-diiPc/FP07 at the same dosage. The optimal concentration recommended for potential application is 0.01 wt.%. It is noteworthy that formulations Co-2EH/TI870 exhibited a higher tendency to overdose, as evident by the high values of τ_2_ and τ_3_ at concentrations of 0.01 wt.%. This is ascribed to different fatty acid patterns, which results, in the case of cobalt-based drier, in faster drying.

Test coatings of high-solid binders, treated by Fe-diiPc, show significantly lower final hardness (*H*_rel;100d_) than those treated by Co-2EH (Table 2). Initial fast rise of the coating hardness, documented by the *H*_rel;10d_ value, is followed by stagnation while coatings treated by Co-2EH continue in slow hardening up to final value (*H*_rel;100d_). Such difference documents the lower long-term activity of Fe-diiPc. Nevertheless, lower hardness of the films containing Fe-diiPc is not necessarily an obstacle for particular application because it only has a minor effect on their chemical resistance, as evidenced by the MEK test (Table 3). Results of the testing, performed on the coatings cured for 105 days, reveal only a minor effect of drier composition and drier concentration on the resistance time. The experimental data, summarized in Table 3, further prove that thickness of the film has a considerably stronger effect as on MEK resistance that the drying agent.

### 3.2. Characterization of the Binders by Vibration Spectroscopy

Infrared and Raman spectroscopy was used for detailed characterization of the alkyd resins S471, S622, FP07 and TI870. The spectra were measured from samples of fresh binders and from samples cured for 100 days at room temperature. The drier composition and drier concentration have only a minor effect on the spectrum pattern of the cured samples; therefore, only spectra of those treated by Fe-diiPc are presented below.

**Figure 2:**
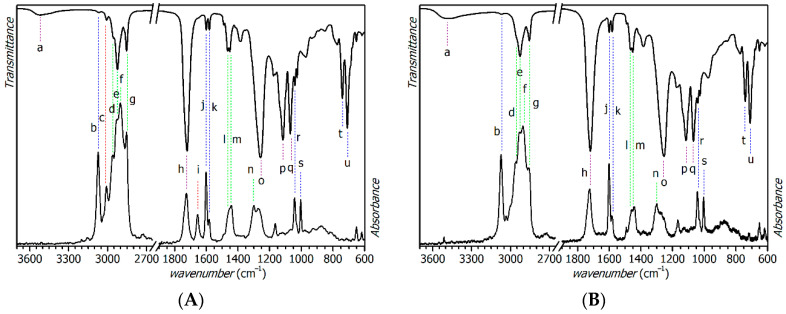
Infrared (top) and Raman spectra (bottom) of S471 formulations: (**A**) after solvent evaporation (no drier used); (**B**) after room temperature curing for 100 days (treated with Fe-diiPc, 0.001 wt.%). Colors: red—bands disappearing upon curing; green—aliphatic chains; blue—aromatic rings; purple—OH and ester functions.

In the binder S471, 21 characteristic absorption bands were observed in the spectra and unambiguously agreed with prior results in the literature [35,36]. They are denoted a–u and are summarized in Table 4. Full spectra are presented in Figure 2. O–H stretching mode (a) of unesterified carboxylic and hydroxyl groups appears in the IR spectrum of the fresh binder as a very broad band at 3520 cm^−1^. Upon the curing process, it moves to lower wavenumbers due to the formation of hydroperoxide by autoxidation of the unsaturated fatty acid chains and appearance of the OH-containing side products [26]. Disappearance of the absorption bands c (IR: 3008 cm^−1^; Raman: 3010 cm^−1^) and i (Raman: 1658 cm^−1^) is the most characteristic change in the vibration spectra of the binder S471.

The bands were assigned to C–H stretching and C=C stretching of the C=C–H moiety in unsaturated fatty acid tails, respectively. We note that both bands can be used for monitoring the unsaturation degree upon the curing process. The absorption bands related with aromatic rings (b, j, k, r, s and t) stayed almost unchanged upon the curing. Only band u, assigned to out-of-plane bending of the C–H bond in the aromatic ring, decreased in intensity due to the superposition of similar vibration modes of the reactive *cis*-C=C–H moiety [37]. Frequency of aromatic ring breathing, intense and well separated in Raman spectra, enables distinguishing between phthalate (band r: 1042 cm^−1^) and isophthalate functions (band s: 1004 cm^−1^). We note that the aforementioned bands b, j and k are very typical for binders containing aromatic rings.

Saturated parts of the hydrocarbon chains give very characteristic C–H stretching bands b–g assigned to methyl and methylene groups. Their decrease in intensity is attributed to oxidative degradation, responsible for the emission of volatile side products, and to a lower flexibility of the fatty acid chains related with a decrease in given extinction coefficients. Ester functions of the polyester backbone give characteristic stretching bands h, o, p and q. Broadening of the C=O stretching band h, which is very strong in infrared spectra and well separated, is attributed to the formation of aldehyde and ketone functions upon the oxidative degradation.

**Figure 3:**
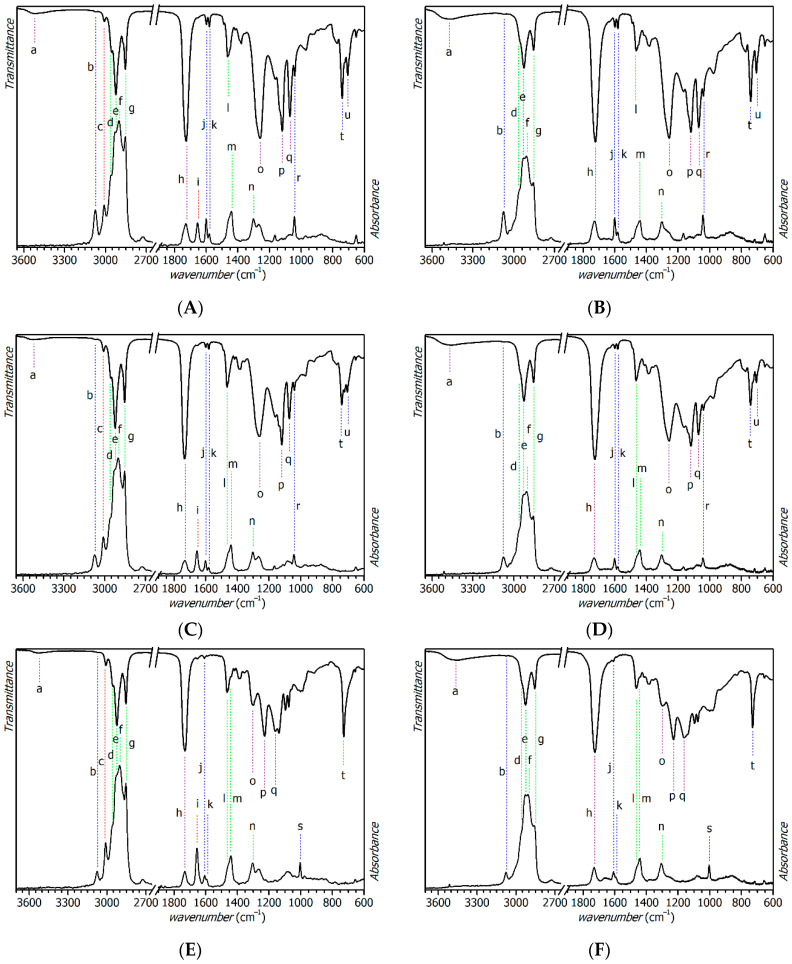
Infrared (top) and Raman spectra (bottom) of formulations: (**A**) fresh S622; (**B**) cured S622; (**C**) fresh FP07; (**D**) cured FP07; (**E**) fresh TI870; (**F**) cured TI870. Colors: red—bands disappearing upon curing; green—aliphatic chains; blue—aromatic rings; purple—ester and OH functions.

Vibration spectra of fresh and cured samples of the resins S622, FP07 and TI870 are presented in Figure 3. Assignments of characteristic absorption bands were performed in line with the binder S471; detailed assignments are given in Tables S2–S4 in the Supplementary Materials.

The infrared and Raman spectra, given in Figure 3, accurately document an effect of oil length on the spectrum pattern. The increasing oil-length of the alkyd binder resulted in a lower relative intensity of the absorption bands related with the fatty acid tails (c–g and i). These bands increased in the order S471 < S622 < FP07 ~ TI870. Lower intensity of the bands b, j and k in the spectra of high-solid binders FP07 and TI870 reflects a lower content of phthalic or isophthalic acid, respectively. As mentioned before, phthalate (S622 and FP07) and isophthalate (TI870) can be distinguished in the binder by breathing of the aromatic ring (ν_s_(C=C, arom.)) active in the Raman spectra (bands r and s). Different patterns in the region of δ(C=C, arom.) are also typical features for this type of modification.

We note that curing of the resins S622, FP07 and TI870 had the same effect in vibration spectra as described for S471. In all cases, the bands c and i were suitable for monitoring the curing process, and composition of the drier had only a minor effect on the intensity of infrared and Raman absorption bands of the cured samples. The only noticeable effect was a higher infrared intensity of bands e and g in the samples treated by Fe-diiPc, when compared with samples treated with Co-2EH. It is ascribed to a higher flexibility of the fatty acid chains resulting in increased extinction coefficients. Suggested lower density of crosslinking is in line with lower relative hardness of the coatings cured by Fe-diiPc documented by the aforementioned mechanical tests.

### 3.3. Investigation of the Autoxidation Kinetics on Test Coatings

Time-resolved infrared spectroscopy was used for a detailed investigation of the curing process. The study was conducted on alkyd binder S471 treated by Fe-diiPc, and the obtained kinetic data were compared with samples treated by Co-2EH. We note that the measurements were performed on thin samples of the formulation using the ATR sampling technique, which prevents formation of inhomogeneities due to limited air-oxygen diffusion into whole paint layer [25].

**Figure 4:**
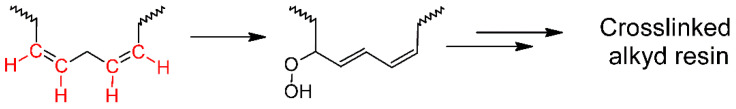
Simplified mechanism of the autoxidation process with highlighted *cis*-C=*C*–*H* groups monitored by infrared spectroscopy.

Consumption of the reactive double allylic moiety, shown in Figure 4, was evidenced in the infrared spectra as the intensity decrease in the absorption band at 3008 cm^−1^, attributed to ν_a_(*cis*-C=C–H) as mentioned in the preceding section (band **c** in the Figure 2a). It should be noted that registered reaction profiles do not reflect the appearance of crosslinks; hydroperoxides are formed as relatively stable intermediates. These, however, are usually decomposed by the action of primary driers as well [11].

**Figure 5:**
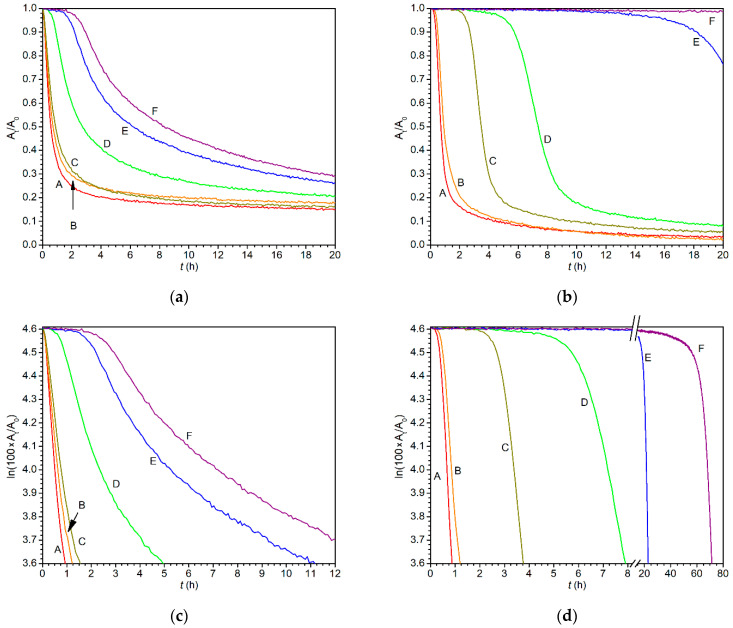
Time development of the infrared band ν_a_(*cis*-C=C–H) of alkyd formulation S471 treated by: (**a**) Fe-diiPc in linear scale; (**b**) Co-2EH in linear scale; (**c**) Fe-diiPc in logarithmic scale; (**d**) Co-2EH in logarithmic scale. Metal concentrations for runs A–F are given in Table 5.

In line with previous studies, the peroxidation step was treated as a reaction of pseudo-first order because the concentration of molecular oxygen is expected to be constant upon the curing process [17,25]. Estimated maximal rate coefficients (*k*_max_), induction times (*t*_ind_) and half-lives (*t*_1/2_) for formulations Fe-diiPc/S471 and Co-2EH/S471 are listed in Table 5. Corresponding reaction profiles are given in Figure 5.

In the concentration range 0.01–0.003 wt.% (runs A–C), diiPc gives very short induction times and high rate coefficients, which correlates well with the short tack-free times (τ_2_) estimated by the mechanical tests. It is noteworthy that commercial Co-2EH gives similar rate coefficients at concentrations of about one magnitude higher; see runs A–C in Table 5. Another important feature of the formulations treated by diiPc is much slower increase in the induction time when the metal concentration decreases. Indeed, very long induction times are not observed even at the concentration 3 × 10^−4^ wt.% (run F), while the limiting dosage of the cobalt-based drier Co-2EH was estimated to be 0.001 wt.% (run D).

Reaction profiles of the formulations Fe-diiPc/S471 and Co-2EH/S471 in linear scale (Figure 5a,b) well document the effect of concentration on the induction time. Furthermore, they show that the coatings containing Fe-diiPc reached significantly lower conversions after 20 h of curing even at the high dosage. Such a phenomenon is related with a deviation from the pseudo-first order kinetics appearing at lower conversions, as became evident from nonlinearity in the logarithmic plots given in Figure 5c (cf. with Figure 5d).

**Figure 6:**
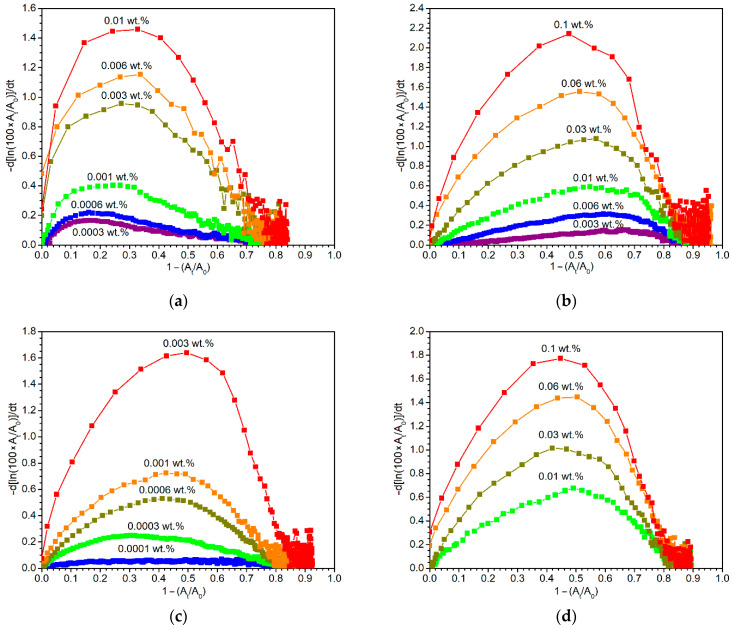
Dependence of rate coefficient (in h^−1^) on conversion for formulations of S471 treated by (**a**) Fe-diiPc; (**b**) Co-2EH; (**c**) Borchi OXY-Coat; (**d**) Mn(acac)_3_.

Different shapes of reaction profiles led us to analyze the experimental data in more detail. It was found that composition of the drier and its concentration have a strong effect not only on the magnitude of maximal rate coefficients (*k*_max_) but also on the conversion at which *k*_max_ is reached. Figure 6 displays the development of the pseudo-first order rate coefficient on the allylic moiety conversion for two driers under study, together with two other promising primary driers, namely Borchi OXY-Coat and Mn(acac)_3_, which will be helpful for better understanding of the phenomenon. We note that the kinetic parameters for Borchi OXY-Coat are given in Table S5 in the Supplementary Materials, while those for Mn(acac)_3_ have been published elsewhere [17].

Figure 6 documents that these four primary driers are able to reach high values of *k*_max_ (>1.4 h^−1^), but each of them at different metal concentrations. From this point of view, the efficiency of the driers increases in the order Mn(acac)_3_ ~ Co-2EH < Fe-diiPc < Borchi OXY-Coat. At high metal concentrations (0.01–0.003 wt.%), Fe-diiPc reaches the maximal value of the rate coefficient at a low conversion (~30%) and the following decrease to a half-maximal value is also relatively fast; it appears at ~60% conversion. At a concentration lower than 0.003 wt.%, the maximal and half-maximal rate coefficients appear at even lower conversions. Such behavior differs considerably from Co-2EH, which, at 0.1 wt.%, exhibits the maximal and half-maximal values of the rate coefficient at ~50% and 75% conversion, respectively. Furthermore, lowering of the metal concentration has an opposite effect on these parameters. The unusual behavior of Fe-diiPc can be ascribed to lower stability of the active species, which is responsible for earlier deviation from pseudo-first order kinetics. Such reasoning also fits the concentration dependence observed for Fe-diiPc. Indeed, slower peroxidation process at a lower metal concentration should lead to the decomposition of higher fractions of catalyst at a given conversion if stability of the active species is the issue. Considering the shape of the plots given in Figure 6, we suggest the stability of the active species increases in the order Fe-diiPc < Borchi OXY-Coat ~ Mn(acac)_3_ ~ Co-2EH.

### 3.4. Coloration of Test Coatings

The red-brown color of solid Fe-diiPc is another issue not discussed in the preliminary studies. Therefore, we decided to evaluate the coloration of formulations Fe-diiPc/S471 and Co-2EH/S471 in concentration ranges used for mechanical tests mentioned before. The measurements were performed in transmission mode on coatings of 200 μm wet thickness.

All coatings under study showed discoloration to greenish-yellow, as documented by data given in Table 6. Deeper coloration of the coatings treated by Fe-diiPc is partially compensated by lower dosages necessary for application. Nevertheless, for full compensation, one should use about 60-fold lower dosage than in the case of Co-2EH, because Fe-diiPc/S471 at 0.001 wt.% exhibits virtually the same chromatic shift as Co-2EH/S471 at 0.06 wt.%. It should be noted that the curing activity of Fe-diiPc is reduced at very low dosage necessary to prevent undesired coloration the transparent coating layers. Therefore, further investigation should be done to improve its curing activity whilst maintaining a low coloration.

## 4. Conclusions

In summary, this study has described the performance of a recently developed iron-based primary drier, Fe-diiPc, in formulations of several alkyd resins commonly used in the paint-producing industry. The main advantage of this trial product is its high activity at considerably lower concentrations than usual for commercially successful cobalt compounds, which became evident mainly on the solvent-borne formulations. Lower activity in the formulations of high-solid binders can be ascribed to their lower polarity that is given by a considerably higher oil length. We note that some differences in performance in binders FP07 and TI870 are due to different fatty acid pattern. Lower relative hardness of the test coatings is the main drawback of Fe-diiPc, because it suggests a low long-term activity. Nevertheless, it could be overcome by combination with another primary drier, given in small quantity, which is a strategy often used in the paint-producing industry.

Kinetic data of the peroxidation process, obtained from time-resolved infrared spectra, proved that the system Fe-diiPc/S471 reaches the rate coefficient of a similar magnitude as Co-2EH/S471, but at a much lower dosage. Such a property is ascribed to the specific coordination environment of the central metal forced by a helmet-shaped phthalocyaninato-like ligand.

Although the overall catalytic activity of Fe-diiPc does not reach the level of promising commercial product Borchi OXY-Coat, its main advantage is a less demanding synthesis from readily available starting material without necessity for its isolation in pure form [10,38]. For practical application in paint systems, one should be aware of the lower final hardness of protective coatings, and their deeper coloration when dosages higher than 0.001 wt.% are used.

## Figures and Tables

**Table 1:** Drying times ^1^ and relative hardness ^2^ of solvent-borne alkyd formulations.

Binder	Drier	C (%)	τ_2_ (h)	τ_3_ (h)	τ_4_ (h)	*H*_rel;10d_ (%)	*H*_rel;100d_ (%)
S471	Fe-diiPc	0.01	0.3	5.2	7.5	19.3	36.6
		0.006	0.5	4.2	5.8	18.6	35.1
		0.003	1.1	2.7	4.3	16.0	31.7
		0.001	8.0	15.5	15.5	23.5	39.9
	Co-2EH	0.1	0.9	6.5	12.5	11.1	41.1
		0.06	2.5	7.0	>24	18.4	46.5
		0.03	9.8	11.3	>24	27.2	47.6
		0.01	>24	>24	>24	14.7	38.9
S622	Fe-diiPc	0.01	0.9	2.6	3.8	9.4	16.9
		0.006	2.0	4.3	6.8	10.8	18.7
		0.003	4.7	8.2	8.2	11.2	18.6
		0.001	9.7	21.7	21.7	9.8	17.0
	Co-2EH	0.1	1.7	7.0	14.7	18.8	39.9
		0.06	2.8	9.0	12.3	17.4	39.0
		0.03	6.6	10.5	12.7	16.8	35.7
		0.01	16.2	19.9	>24	14.0	30.6

^1^ Tack-free time (τ_2_), dry-hard time (τ_3_) and dry-through time (τ_4_); ^2^ Relative hardness was estimated 10 days (*H*_rel;10d_) and 100 days after application (*H*_rel;10d_).

**Table 2:** Drying times ^1^ and relative hardness ^2^ of high-solid alkyd formulations.

Binder	Drier	C (%)	τ_1_ (h)	τ_2_ (h)	τ_3_ (h)	τ_4_ (h)	*H*_rel;10d_ (%)	*H*_rel;100d_ (%)
FP07	Fe-diiPc	0.01	1.5	3.9	8.3	8.3	6.6	7.6
		0.006	2.3	5.5	11.4	>24	6.5	7.1
		0.003	5.2	14.5	>24	>24	6.9	7.8
		0.001	13.0	>24	>24	>24	– ^3^	– ^3^
	Co-2EH	0.1	0.8	2.5	20.6	>24	8.8	16.4
		0.06	0.8	3.0	12.0	>24	7.4	13.5
		0.03	1.6	4.7	11.7	>24	6.5	11.9
		0.01	2.7	4.9	11.3	14.7	5.9	9.1
TI870	Fe-diiPc	0.01	2.7	8.2	11.9	>24	6.4	6.9
		0.006	5.1	8.0	18.0	>24	6.2	6.7
		0.003	12.9	>24	>24	>24	6.8	7.2
		0.001	>24	>24	>24	>24	– ^3^	– ^3^
	Co-2EH	0.1	0.5	3.3	>24	>24	10.7	25.4
		0.06	0.7	2.6	15.3	>24	9.1	21.8
		0.03	1.2	4.3	12.6	>24	6.9	17.9
		0.01	2.7	4.0	7.8	>24	6.5	13.0

^1^ Set-to-touch time (τ_1_), tack-free time (τ_2_), dry-hard time (τ_3_) and dry-through time (τ_4_); ^2^ Relative hardness estimated 10 days (*H*_rel;10d_) and 100 days after application (*H*_rel;10d_); ^3^ Not measured owing to slow drying.

**Table 3:** MEK resistance for a given alkyd binder ^1^.

Drier	C (%)	S471	S622	FP07	TI870
Fe-diiPc	0.01	42 (44)	39 (50)	43 (29)	40 (26)
	0.006	39 (49)	50 (38)	58 (31)	49 (32)
	0.003	50 (61)	51 (37)	38 (25)	46 (27)
	0.001	37 (33)	58 (51)	– ^2^	– ^2^
Co-2EH	0.1	50 (64)	42 (40)	39 (33)	47 (32)
	0.06	53 (46)	57 (38)	40 (30)	52 (30)
	0.03	46 (45)	46 (38)	47 (39)	51 (36)
	0.01	59 (62)	48 (41)	45 (38)	48 (23)

^1^ Resistance time (in seconds). Film thicknesses (in μm) are given in parentheses. ^2^ Not measured owing to slow drying of the formulation.

**Table 4:** Assigned characteristic vibration modes for binder **S471** ^1^.

Sign	IR	Raman	IR	Raman	Assignment
	Fresh sample		Cured sample		
a	3520 m-br	–	3490 m-br	–	ν(O–H)
b	3068 vw	3073 s	3071 vw	3074 s	ν(C–H, arom.)
c	3008 w	3010 w	–	–	ν_a_(*cis*-C=C–H)
d	2954 wv	2961 vw	2958 sh	2958 vw	ν_a_(C–H, CH_3_)
e	2924 s	2930 vw	2927 m	2934 vw	ν_a_(C–H, CH_2_)
f	–	2901 vs	–	2904 vs	ν_s_(C–H, CH_3_)
g	2854 m	2854 w	2855 w	2858 vw	ν_s_(C–H, CH_2_)
h	1723 vs	1727 m	1720 vs	1725 m	ν(C=O)
i	–	1657 m	–	–	ν(*cis*-C=C–H)
j	1600 w	1602 s	1600 w	1602 s	ν(C=C, arom.)
k	1580 w	1582 w	1581 w	1583 w	ν(C=C, arom.)
l	1466 m	1455 sh	1465 m	1454 vw	δ(C–H, CH_3_/CH_2_)
m	1451 m	1442 m	1451 m	1444 m	δ(C–H, CH_3_/CH_2_)
n	–	1301 m	–	1300 m	δ(C–H, CH_2_)
o	1258 vs	–	1254 vs	–	ν(C–O, ester)
p	1116 s	–	1115 s	–	ν(C–O, ester)
q	1070 s	–	1068 s	–	ν(C–O, ester)
r	1040 w	1042 m	1041 w	1043 m	ν_s_(C=C, arom., 1,2-disubst.)
s	–	1004 m	–	1005 m	ν_s_(C=C, arom., 1,3-disubst.)
t	741 s	–	741 s	–	δ(C–H, arom.)
u	710 s	–	711 s	–	δ(C=C, arom.)/δ(*cis*-C=C–H)

^1^ Wavenumbers are given in cm^−1^; intensity of the absorption bands is expressed as follows: br, broad; m, medium; s, strong; sh, shoulder; vs, very strong; vw, very weak.

**Table 5:** Kinetic parameters for formulations of the binder **S471** ^1^.

Drier	Run	C (%)	*t*_ind_ (h)	*k*_max_ (h^−1^)	*t*_1/2_ (h)
Fe-diiPc	A	0.01	<0.1	1.43	0.6
	B	0.006	<0.1	1.12	0.7
	C	0.003	0.1	0.94	0.9
	D	0.001	0.6	0.40	2.7
	E	0.0006	1.7	0.22	6.2
	F	0.0003	2.3	0.17	8.4
Co-2EH	A	0.1	0.4	2.12	0.7
	B	0.06	0.5	1.55	0.9
	C	0.03	2.8	1.08	3.4
	D	0.01	6.1	0.57	7.4
	E	0.006	19.7	0.32	21.9
	F	0.003	64.2	0.15	69.0

^1^ Data for peroxidation step determined by time-resolved infrared spectroscopy.

**Table 6:** Coloration of transparent coatings treated by Fe-diiPc and Co-2EH ^1^.

Drier	C (wt.%)	*L	*a	*b
Fe-diiPc	0.01	96.2	−0.81	5.65
	0.006	97.5	−0.40	2.97
	0.003	98.0	−0.18	1.64
	0.001	99.1	−0.08	0.64
Co-2EH	0.1	99.0	−0.17	1.06
	0.06	98.9	−0.11	0.70
	0.03	99.7	−0.09	0.52
	0.01	99.7	−0.11	0.46

^1^ Formulations of S471 collected 3 days after application on glass substrate. Wet thickness: 200 μm.

## Data Availability

The data presented in this study are available on request from the corresponding author.

## Supplementary Materials

The following are available online at https://www.mdpi.com/1996-1944/14/5/1220/s1, Table S1: Drying times for formulations of Borchi OXY-Coat in S471, Table S2: Assigned characteristic vibration modes for binder S622, Table S3: Assigned characteristic vibration modes for binder FP07, Table S4: Assigned characteristic vibration modes for binder TI870, Table S5: Kinetic parameters for formulation Borchi OxyCoat/S471.

## References

[1] Bottari G., de la Torre G., Guldi D.M., Torres T. (2010). Covalent and Noncovalent Phthalocyanine−Carbon Nanostructure Systems: Synthesis, Photoinduced Electron Transfer, and Application to Molecular Photovoltaics. Chem. Rev..

[2] Sorokin A.B. (2013). Phthalocyanine metal complexes in catalysis. Chem. Rev..

[3] Kieler H.M., Bierman M.J., Guzei I.A., Liska P.J., McGaff R.W. (2006). Racemic iron(III) and cobalt(III) complexes containing a new pentadente “helmet” phthalocyaninato ligand. Chem. Commun..

[4] Brash A.R. (1999). Lipoxygenases: Occurrence, functions, catalysis, and acquisition of substrate. J. Biol. Chem..

[5] Skobelev I.Y., Kudrik E.V., Zalomaeva O.V., Albrieux F., Afanasiev P., Kholdeeva O.A., Sorokin A.B. (2013). Efficient epoxidation of olefins by H_2_O_2_ catalyzed by iron “helmet” phthalocyanines. Chem. Commun..

[6] McGaff R.W. (2017). Bridged Phthalocyanine-and Napththalocyanine—Metal Complex Catalysts and Methods of Using and Purifying the Same. US Patent.

[7] Brown E.S., Robinson J.R., McCoy A.M., McGaff R.W. (2011). Efficient catalytic cycloalkane oxidation employing a “helmet” phthalocyaninato iron(III) complex. Dalton Trans..

[8] Peterson B.M., Herried M.E., Neve R.L., McGaff R.W. (2014). Oxidation of primary and secondary benzylic alcohols with hydrogen peroxide and tert-butyl hydroperoxide catalyzed by a “helmet” phthalocyaninato iron complex in the absence of added organic solvent. Dalton Trans..

[9] Neve R.L., Eidenschink M.C., Guzei I.A., Peterson B.M., Vang G.M., McGaff R.W. (2016). Homogeneous catalytic oxidation of unactivated primary and secondary alcohols employing a versatile “Helmet” phthalocyaninato iron complex catalyst without added organic solvent. ChemistrySelect.

[10] Dubrulle L., Lebeuf R., Nardello-Rataj V. (2019). Oxidative drying properties of a helmet pentadentate phthalocyanine-derived iron(III) complex. Prog. Org. Coat..

[11] Honzíček J. (2019). Curing of Air-Drying Paints: A Critical Review. Ind. Eng. Chem. Res..

[12] Hofland A. (2012). Alkyd resins: From down and out to alive and kicking. Prog. Org. Coat..

[13] Leyssens L., Vinck B., Van Der Straeten C., Wuyts F., Maes L. (2017). Cobalt toxicity in humans—A review of the potential sources and systemic health effects. Toxicology.

[14] Simpson N., Maaijen K., Roelofsen Y., Hage R. (2019). The Evolution of Catalysis for Alkyd Coatings: Responding to Impending Cobalt Reclassification with Very Active Iron and Manganese Catalysts, Using Polydentate Nitrogen Donor Ligands. Catalysts.

[15] Bouwman E., van Gorkum R. (2007). A study of new manganese complexes as potential driers for alkyd paints. J. Coat. Technol. Res..

[16] Wu J.Z., Bouwman E., Reedijk J. (2004). Chelating ligands as powerful additives to manganese driers for solvent-borne and water-borne alkyd paints. Prog. Org. Coat..

[17] Matušková E., Honzíček J. (2020). Performance of Manganese(III) Acetylacetonate in Solvent-Borne and High-Solid Alkyd Formulations. Materials.

[18] Preininger O., Honzíček J., Kalenda P., Vinklárek J. (2016). Drying activity of oxovanadium(IV) 2-ethylhexanoate in solvent-borne alkyd paints. J. Coat. Technol. Res..

[19] Charamzová I., Honzíček J., Kalenda P., Vinklárek J., Císařová I. (2020). Dimeric Oxidovanadium(IV) Complex Bearing 1,10-Phenanthroline. Crystallogr. Rep..

[20] Preininger O., Vinklárek J., Honzíček J., Mikysek T., Erben M. (2015). A promising drying activity of environmentally friendly oxovanadium(IV) complexes in air-drying paints. Prog. Org. Coat..

[21] Preininger O., Charamzová I., Vinklárek J., Císařová I., Honzíček J. (2017). Oxovanadium(IV) complexes bearing substituted pentane-2,4-dionate ligands: Synthesis, structure and drying activity in solvent-borne alkyd paints. Inorg. Chim. Acta.

[22] Charamzová I., Machálková A., Vinklárek J., Císařová I., Honzíček J. (2019). Benzyl substituted oxidovanadium (IV) pentane-2, 4-dionates: Synthesis, structure and drying properties. Inorg. Chim. Acta.

[23] Charamzová I., Vinklárek J., Kalenda P., Císařová I., Honzíček J. (2020). Oxidovanadium(V) dithiocarbamates as driers for alkyd binders. J. Coat. Technol. Res..

[24] de Boer J.W., Wesenhagen P.V., Wenker E.C., Maaijen K., Gol F., Gibbs H., Hage R. (2013). The Quest for Cobalt-Free Alkyd Paint Driers. Eur. J. Inorg. Chem..

[25] Charamzová I., Vinklárek J., Honzíček J. (2018). Effect of primary driers on oxidative drying of high-solid alkyd binder: Investigation of thickness effects by mechanical tests and infrared spectroscopy. Prog. Org. Coat..

[26] Křižan M., Vinklárek J., Erben M., Císařová I., Honzíček J. (2017). Autoxidation of alkyd resins catalyzed by iron(II) bispidine complex: Drying performance and in-depth infrared study. Prog. Org. Coat..

[27] Křižan M., Vinklárek J., Erben M., Růžičková Z., Honzíček J. (2019). Iron(II) complex with modified bispidine ligand: Synthesis and catalytic alkyd drying. Inorg. Chim. Acta.

[28] Erben M., Veselý D., Vinklárek J., Honzíček J. (2012). Acyl-substituted ferrocenes as driers for solvent-borne alkyd paints. J. Mol. Catal. A Chem..

[29] Honzíček J., Fedorova T., Vinklárek J., Mikysek T., Císařová I. (2020). Modified Ferrocenes as Primary Driers for Formulations of Alkyd Paints. Coatings.

[30] AKZO Nobel Coatings International B.V. (2021). Coating Composition Comprising an Autoxidizable Resin and an Iron–ligand Complex, Substrate Coated with Such Coating Composition, and Use of Such Iron–ligand complex. WO Patent.

[31] ASTM D5895-20 (2020). Standard Test Methods for Evaluating Drying or Curing During Film Formation of Organic Coatings Using Mechanical Recorders.

[32] ISO 1522:2006 (2007). Paints and Varnishes—Pendulum Damping Test.

[33] ASTM D4752-20 (2020). Standard Practice for Measuring MEK Resistance of Ethyl Silicate (Inorganic) Zinc-Rich Primers by Solvent Rub.

[34] ISO 2808:2007 (2007). Paints and Varnishes—Determination of Film Thickness.

[35] Socrates G. (2001). Infrared and Raman Characteristic Group Frequencies: Tables and Charts.

[36] Ellis G., Claybourn M., Richards S.E. (1990). The application of Fourier transform Raman spectroscopy to the study of paint systems. Spectrochim. Acta Part A.

[37] De Viguerie L., Payard P.A., Portero E., Walter P., Cotte M. (2016). The drying of linseed oil investigated by Fourier transform infrared spectroscopy: Historical recipes and influence of lead compounds. Prog. Org. Coat..

[38] Börzel H., Comba P., Hagen K.S., Lampeka Y.D., Lienke A., Linti G., Merz M., Pritzkow H., Tsymbal L.V. (2002). Iron coordination chemistry with tetra-, penta- and hexadentate bispidine-type ligands. Inorg. Chim. Acta.

