# Supplementary material for: Helmet Phthalocyaninato Iron Complex as a Primary Drier for Alkyd Paints
Source: Materials (Basel). 2021 Mar 5;14(5):1220. doi: 10.3390/ma14051220 (PMC7961973; doi:10.3390/ma14051220)
Supplement: Supplementary file 1 [file materials-14-01220-s001.pdf]

materials

Supplementary Material

Helmet Phthalocyaninato Iron Complex as a Primary Drier for Alkyd Paints

Jan Honzíček [1,*], Eliška Matušková [1], Štěpán Voneš [1] and Jaromír Vinklárek [2]

[1] Institute of Chemistry and Technology of Macromolecular Materials, Faculty of Chemical Technology, University of Pardubice, Studentská 573, 532 10 Pardubice, Czech Republic
[2] Department of General and Inorganic Chemistry, Faculty of Chemical Technology, University of Pardubice, Studentská 573, 532 10 Pardubice, Czech Republic
* Correspondence: jan.honzicek@upce.cz; Tel.: +420-466-037-229

Citation: Honzíček, J.; Matušková, E.; Voneš, Š.; Vinklárek, J. Helmet Phthalocyaninato Iron Complex as a Primary Drier for Alkyd Paints. *Materials* **2021**, *14*, 1220. https://doi.org/10.3390/ma14051220

Academic Editor: Barbara Pawelec

Received: 12 February 2021
Accepted: 02 March 2021
Published: 5 March 2021

Publisher's Note: MDPI stays neutral with regard to jurisdictional claims in published maps and institutional affiliations.

Copyright: © 2021 by the authors. Licensee MDPI, Basel, Switzerland. This article is an open access article distributed under the terms and conditions of the Creative Commons Attribution (CC BY) license (http://creativecommons.org/licenses/by/4.0/).

Table S1. Drying times for formulations of Borchi OXY-Coat in S471.[1]

C (%)	τ_2 (h)	τ_3 (h)	τ_4 (h)
0.003	0.3	7.8	13.0
0.001	0.8	2.8	9.6
0.0006	1.7	3.8	6.7
0.0003	3.2	6.0	6.0
0.0001	8.6	>24	>24

[1] Tack-free time (τ_2), dry-hard time (τ_3) and dry-through time (τ_4).

Table S2. Assigned characteristic vibration modes for binder S622.

Sign	IR	Raman	IR	Raman	Assignment
	Fresh sample		Cured sample		
a	3525 w-br	–	3457 m-br	–	ν(O–H)
b	3070 vw	3074 m	3074 vw	3075 m	ν(C–H, arom.)
c	3008 w	3009 w	–	–	ν_a(*cis*-C=C–H)
d	2954 vw	2958 sh	2955 sh	2958 sh	ν_a(C–H, CH_3)
e	2923 s	2928 vw	2926 m	2928 vw	ν_a(C–H, CH_2)
f	–	2901 vs	–	2906 s	ν_s(C–H, CH_3)
g	2853 m	2854 w	2855 w	2857 w	ν_s(C–H, CH_2)
h	1728 vs	1731 m	1724 vs	1730 m	ν(C=O)
i	–	1657 m	–	–	ν(*cis*-C=C–H)
j	1599 w	1601 m	1599 w	1601 m	ν(C=C, arom.)
k	1580 w	1581 w	1580 w	1581 w	ν(C=C, arom.)
l	1465 m	–	1465 m	–	δ(C–H, CH_3/CH_2)
m	–	1442 m	–	1441 m	δ(C–H, CH_3/CH_2)
n	–	1302 m	–	1301 m	δ(C–H, CH_2)
o	1259 vs	–	1255 vs	–	ν(C–O, ester)
p	1119 vs	–	1119 s	–	ν(C–O, ester)
q	1070 s	–	1069 s	–	ν(C–O, ester)
r	1040 w	1042 m	1040 w	1043 m	ν_s(C=C, arom., 1,2-disubst.)
t	741 s	–	741 s	–	δ(C–H, arom.)
u	705 w	–	705 w	–	δ(C=C, arom.)/δ(*cis*-C=C–H)

Table S3. Assigned characteristic vibration modes for binder FP07.

Sign	IR	Raman	IR	Raman	Assignment
	Fresh sample		Cured sample		
a	3524 w-br	–	3468 m-br	–	ν(O–H)
b	3068 vw	3074 w	3071 vw	3075 w	ν(C–H, arom.)
c	3008 w	3010 w	–	–	$ν_a$(*cis*-C=C–H)
d	2954 vw	2959 sh	2957 sh	2958 sh	$ν_a$(C–H, CH_3)
e	2923 s	2926 sh	2926 m	2928 vw	$ν_a$(C–H, CH_2)
f	–	2901 vs	–	2905 s	$ν_s$(C–H, CH_3)
g	2853 m	2854 w	2855 w	2857 vw	$ν_s$(C–H, CH_2)
h	1735 vs	1734 w	1727 vs	1732 w	ν(C=O)
i	–	1657 m	–	–	ν(*cis*-C=C–H)
j	1600 vw	1602 w	1601 vw	1601 w	ν(C=C, arom.)
k	1580 vw	1581 vw	1580 vw	1579 vw	ν(C=C, arom.)
l	1465 m	1458 sh	1465 m	1461 sh	δ(C–H, CH_3/CH_2)
m	–	1441 m	–	1441 m	δ(C–H, CH_3/CH_2)
n	–	1302 m	–	1306 m	δ(C–H, CH_2)
o	1259 s	–	1256 s	–	ν(C–O, ester)
p	1119 s	–	1119 s	–	ν(C–O, ester)
q	1072 m	–	1071 m	–	ν(C–O, ester)
r	1040 w	1042 w	1040 w	1043 w	$ν_s$(C=C, arom., 1,2-disubst.)
t	740 m	–	742 m	–	δ(C–H, arom.)
u	705 vw	–	705 vw	–	δ(C=C, arom.)/δ(*cis*-C=C–H)

Table S4. Assigned characteristic vibration modes for binder TI870.

Sign	IR	Raman	IR	Raman	Assignment
	Fresh sample		Cured sample		
a	3526 w-br	–	3460 m-br	–	ν(O–H)
b	3068 vw	3076 w	3074 vw	3077 w	ν(C–H, arom.)
c	3008 w	3010 w	–	–	$ν_a$(*cis*-C=C–H)
d	2953 vw	2961 sh	2955 sh	2961 sh	$ν_a$(C–H, CH_3)
e	2924 s	2928 sh	2926 m	2928 s	$ν_a$(C–H, CH_2)
f	–	2903 vs	–	2907 s	$ν_s$(C–H, CH_3)
g	2854 m	2854 w	2855 w	2860 vw	$ν_s$(C–H, CH_2)
h	1732 vs	1732 w	1727 vs	1732 w	ν(C=O)
i	–	1657 m	–	–	ν(*cis*-C=C–H)
j	1608 vw	1606 vw	1609 vw	1607 w	ν(C=C, arom.)
k	–	1593 vw	–	1593 vw	ν(C=C, arom.)
l	1466 m	1463 sh	1464 m	1461 sh	δ(C–H, CH_3/CH_2)
m	1438 sh	1441 m	1438 sh	1441 m	δ(C–H, CH_3/CH_2)
n	–	1304 m	–	1307 m	δ(C–H, CH_2)
o	1299 m	–	1298 w	–	ν(C–O, ester)
p	1227 s	–	1228 s	–	ν(C–O, ester)
q	1161 s	–	1162 s	–	ν(C–O, ester)
s	–	1004 w	–	1004 w	$ν_s$(C=C, arom., 1,3-disubst.)
t	728 s	–	729 s	–	δ(C–H, arom.)

Table S5. Kinetic parameters for formulation Borchi OxyCoat/S471.

C (%)	t_{ind} (h)	k_{max} (h^{-1})	$t_{1/2}$ (h)
0.003	0.3	1.64	0.8
0.001	0.8	0.70	1.8
0.0006	1.3	0.53	2.7
0.0003	2.3	0.25	5.3
0.0001	5.4	0.06	18.2
